# The Roles of Individual and Psychosocial Factors in Predicting Quality of Life Among Working Women in Shanghai

**DOI:** 10.3390/ijerph17051751

**Published:** 2020-03-07

**Authors:** Yi Xiao, Tao Zhang, Xiangli Gu, Joonyoung Lee, Hongying Wang

**Affiliations:** 1 China Table Tennis College, Shanghai University of Sport, Shanghai 200438, China; teacherxy@sus.edu.cn; 2 Department of Kinesiology, Health Promotion and Recreation, University of North Texas, Denton, TX 76203, USA; joonyounglee@my.unt.edu; 3 Department of Kinesiology, University of Texas at Arlington, Arlington, TX 76019, USA; Xiangli.Gu@uta.edu; 4 School of Leisure Sport, Shanghai University of Sport, Shanghai 200438, China

**Keywords:** health status, occupational stress, burnout, quality of life, working women, China

## Abstract

Working women are at a high risk of suffering from occupational stress and burnout, which can result in reducing Quality of Life (QoL). Guided by the QoL construct and Luban et al.’s conceptual framework, this study aimed to (a) investigate the roles of individual factors (i.e., age) and psychosocial factors (i.e., occupational stress, burnout) on QoL among working women, and (b) examine the age differences among study variables (young versus middle-aged groups). Participants were 375 working women (*M*_age_ = 42.06) recruited in Shanghai, China. They completed previously validated questionnaires assessing their occupational stress, burnout (emotional exhaustion, cynicism, and reduced professional efficacy), and QoL (physical health, psychological health, social relationship, and living environment). Confirmatory factor analysis, Pearson product-moment correlation, hierarchical regressions, and factorial multivariate analyses of variance (MANOVA) were used to examine the relationships and differences between occupational stress, burnout, and QoL among working women. Correlation and regression analyses indicated that occupational stress and burnout were significantly associated with QoL among these participants. Two one-factor MANOVAs demonstrated that young-aged working women had higher occupational stress and burnout, but lower levels of QoL than middle-aged women. These results suggest that adopting specific coping strategies to reduce or prevent occupational stress and burnout are needed to improve QoL among working women.

## 1. Introduction

Shanghai is one of the most developed cities and the economic center of China, where the life pace is increasingly accelerating and is full of competitions in the working environment. Research has reported that the prevalence of occupational stress is negatively correlated with health-related quality of life (QoL), which is influenced by the work-related environment, ranging from 8.5% to 26.4% (i.e., workload, colleagues and supervisor [1]). Working women in Shanghai (~7.52 million) are a focus of this study and have been identified as an at-risk occupational group because previous studies indicate that they are more likely to perceive more occupational stress and burnout compared to male counterparts [2,3,4].

It was also noted that working women usually suffer from more occupational stress than their male counterparts because of insufficient social support, perceived discrimination, and reduced professional efficacy [5,6]. Occupational stress is the harmful physical and emotional responses that occur when a worker’s capabilities, resources, or needs do not match the job demands [7]. Occupational stress results from a perceived imbalance between workplace stressors and coping abilities of the worker, leading to negative mental health outcomes and low QoL [1,7,8,9,10,11].

According to Wright and Bonnett [12], burnout is identified as a syndrome of emotional exhaustion, cynicism, and lack of professional efficacy. Specifically, emotional exhaustion implies individual feelings of being psychologically overextended and depleted while in contact with other people, cynicism refers to a negative response toward other people and a lack of interest in work, and reduced professional efficacy refers to the degree of one’s feelings of incompetence and unsuccessful performance in one’s work [12,13]. Burnout can not only seriously affect workers’ enthusiasm, satisfaction, innovation, happiness index, but also endanger workers’ physical and mental health, such as irritability, insomnia, memory loss, and loss of appetite [11].

QoL is a comprehensive multidimensional construct that includes individuals’ physical health, psychosocial health, social relationships, and living environment affected by individual beliefs, experiences, and expectations [14,15]. Specifically, physical health implies the physical functional status, such as activities of daily living, dependence on medicinal substances, and mobility. Psychological health refers to psychological wellbeing, such as bodily image and appearance, negative or positive feelings, and self-esteem. Social relationship includes personal relationships and social support. Environment refers to the living circumstances, including access to services, such as financial resources, health and social care, and physical environment. QoL is of growing interest in public health as a general health outcome [14,15,16]. In the health-related QoL construct developed by Ferrans and colleagues [8], characteristics of the individual and environment can affect the functional status of health-related QoL. For example, severe impairments of functional status might interfere with daily occupational stress or activities.

In recent years, occupational stress and burnout have become recognized as global health concerns among various professionals due to continued exposure to a large number of work-related stimuli [4,17,18,19]. Previous studies have been conducted to measure occupational stress levels in certain professions, such as industry employees, teachers, and health professions [9,10,20]. It was documented that individual factors (i.e., gender, age, marriage status) are associated with occupational stress and burnout, which will consequently impact their QoL [11,21]. Research found that female participants experienced symptoms of occupational stress and burnout more often than male employees [22]. The longitudinal evidence also supports that high levels of burnout result in more physical health complaints among social workers [23]. Moreover, women had lower scores in most subscales of QoL than men, and QoL was negatively associated with age [24]. However, more research is needed to examine the age differences among occupational stress, burnout, and QoL among working women [1]. To date, no study has been solely focused on working women in Shanghai and how their occupational stress and burnout may contribute to QoL.

Studies have investigated the relationship between occupational stress and QoL targeted in certain professionals, such as teachers and nurses [12,20]. Yang and colleagues [25], for instance, indicated that male teachers perceived better QoL than female teachers, and QoL deteriorated with age. Another study also showed that individuals with intense occupational stress reported negative life satisfaction, while it had a positive association with the three dimensions of burnout, including emotional exhaustion, lack of personal accomplishment, and depersonalization among accountants in Turkey [18]. Wang and colleagues [20] examined the relationship of occupational stress with job burnout and QoL among female health professionals. They found that occupational stress, role overload, and physical environment were important risk factors for job burnout and QoL among health professionals.

Consistent with a recent conceptual framework [26], a psychosocial mechanism was proposed to understand how interactions between individual factors (i.e., sex, age, weight status) and psychosocial factors (i.e., self-perception, mood, and emotions) may influence mental health outcomes such as QoL. Research focused on the individual and psychosocial correlates or determinants of QoL may help us understand and monitor their mental health status among working women in a high economic pressure city. Studying the QoL of working women is relevant to public health, as the foundation for QoL and the functional status of health are formed early in life. Several previous studies have reported significant improvements in QoL (i.e., physical and psychological well-being) in young adults associated with physical activity [27].

Negative affective experiences can become critical health concerns in the working environment. Understanding whether both occupational stress and burnout are related to individual physical and psychosocial well-being has important public health implications [16]. These research efforts may provide the valuable insight to improve QoL by developing effective coping strategies with regard to occupational stress and burnout. Guided by the QoL construct and Luban et al.’s [26] conceptual framework, the primary purpose of this study is to investigate the roles of individual factors (i.e., age) and psychosocial factors (i.e., occupational stress, burnout) on QoL among working women. It is hypothesized that occupational stress and burnout would be negatively related to QoL among working women in Shanghai. The second purpose is to examine the age differences among study variables (young versus middle-aged groups). It is hypothesized that young women would have higher levels of occupational stress and burnout but lower levels of QoL than middle-aged women.

## 2. Methods

### 2.1. Participants

The study design was cross-sectional, and participants were working women randomly recruited from eight communities in Shanghai, China using the stratified cluster sampling method. The participants were either friend-referrals or self-referrals based on advertisement and recruitment events in communities. The inclusion criteria included female with full-time job, married, with at least one year of working experience, and age ranged from 18 to 60 years old. Specifically, our research assistants went to local communities during their health event programs to recruit the participants. Individuals who expressed willingness to participate in the study completed questionnaires. A total of 400 participants were initially recruited, and 25 individuals were excluded from the data analyses because they did not meet the selection criteria for this study.

According to World Health Organization guidelines and previous literature [21], participants were classified into two age groups: those aged 44 years or younger were classified as the young group, and those aged 45–60 years were classified as the middle-aged group in this study [28]. The study was conducted in accordance with the Declaration of Helsinki, and the approval to conduct the study protocol was obtained from the university institutional approval in Shanghai before the data collection (Project identification code: 16936). All participants signed written consent forms before they joined this study.

### 2.2. Procedure

The participants who signed the consent form were given a full explanation about the study’s purpose, the potential benefits and risks, as well as the confidentiality and withdrawal rights. After that, they were directed to complete the self-reported questionnaires, including occupational stress, burnout, and QoL. These questionnaires were translated from English to Chinese and back-translated from Chinese to English by four independent bilingual translators. The original English and back-translation versions were compared in order to ensure the validity of the questionnaires, and all discrepancies were reviewed and accepted by four independent bilingual translators. Furthermore, we sent the Chinese versions of all questionnaires to a small group of working women for a review in order to get acceptable content-related validity. We also offered a training session for research assistants on how to administer the questionnaires appropriately. To minimize participants’ tendency to give socially desirable responses, they were encouraged to answer the questions truthfully and independently. Participants spent approximately 20–30 min completing the questionnaires.

### 2.3. Measures

#### 2.3.1. Demographic variables

Participants self-reported the basic demographic information, including age and marital status.

#### 2.3.2. Occupational stress

The previously-validated Chinese version of the Psychosomatic Tension and Relaxation Inventory (PSTRI) was used to measure occupational stress in this study [29]. This inventory consists of 50 items rated on a five-point Likert scale ranging from 0 (*never*) to 4 (*always*). The mean score of the 50 items (possible range: 0–4) was used to measure occupational stress. The Cronbach’s alpha of this scale was 0.95 in the current study.

#### 2.3.3. Burnout

The 15-item Maslach Burnout Inventory—General Survey (MBI-GS) was used to assess job burnout [30]. The MBI-GS measures three subscales of burnout: emotional exhaustion, cynicism, and reduced professional efficacy. The example items are “Working with people all day is really a strain for me (emotional exhaustion),” “I doubt the significance of my work (cynicism),” and “I have accomplished many worthwhile things in my job” (reduced professional efficacy; reverse item [13,31]). All these items were rated on a seven-point Likert scale, ranging from 1 (*never*) to 7 (*every day*). The mean scores of each subscale (possible range: 1–7) were used to measure the emotional exhaustion, cynicism, and reduced professional efficacy. Higher scores on the subscales of emotional exhaustion, cynicism, and reduced professional efficacy indicated higher levels of burnout. The Chinese version of the MBI-GS has demonstrated good reliability and validity and has been widely used in the Chinese population [31]. In this study, the Cronbach’s alpha coefficients of emotional exhaustion, cynicism, and reduced professional efficacy were 0.75, 0.71, and 0.74, respectively.

#### 2.3.4. Quality of life

QoL was measured by the Chinese version of the Quality of Life Scale—Brief (WHOQOL-BREF; [32,33]). It is an abbreviated version of the WHOQOL-100 used for assessing the generic quality of life. The WHOQOL-BREF includes 26 items with 4 subscales: physical health, psychological health, social relationship, and environment. The sample items are: “Do you have enough energy for everyday life (physical health)? (1 = *not at all*, 5 = *completely*)”, “How much do you enjoy life (psychological health)? (1 = *not at all*, 5 = *extremely*)”, “How satisfied are you with the support you get from your friends (social relationship)? (1 = *not at all*, 5 = *very satisfied*)”, and “How satisfied are you with your access to health services (environment)? (1 = *not at all*, 5 = *very satisfied*)”. Based on the scoring recommendations of the WHOQOL-BREF, the mean scores of each subscale multiplied by four (possible range: 4–20) were used to measure the physical health, psychological health, social relationship, and environment, respectively [33]. In the present study, the Cronbach’s alpha coefficients of physical health, psychological health, social relationship, and environment were 0.74, 0.71, 0.75, and 0.71, respectively.

### 2.4. Data Analyses

The Statistical Package of the Social Sciences (SPSS 25.0, IBM Corp., Armonk, NY, USA) was used to analyze the data. First, after checking the normality of the data, descriptive statistics were calculated for all variables. Second, Cronbach’s alpha coefficients and composite reliability (CR) were computed to assess the internal consistency and reliability for each scale. Third, a confirmatory factor analysis (CFA) was conducted to test the validity of study variables using AMOS 25.0 (IBM Corp., Armonk, NY, USA) [34]. The model goodness-of-fit was determined as good using the following criteria [35]: the chi-squared test, incremental fit index (IFI; ˃0.90), comparative fit index (CFI; ˃0.90), and root mean square error of approximation (RMSEA; <0.08). Fourth, Pearson product-moment correlations were used to examine the unadjusted associations among occupational stress, burnout (emotional exhaustion, cynicism, and reduced professional efficacy), and four subscales of QoL (physical health, psychological health, social relationship, and environment). Fifth, four hierarchical regressions were performed to assess the relative contribution of individual factor (i.e., age), and psychosocial factors (i.e., occupational stress and burnout) to working women’s four subscales of QoL, respectively. Age was entered in the first block, and occupational stress and burnout were entered second to test the change in the variance accounted for (R^2^ change) at each step. This method was used to determine the relative contribution of occupational stress and burnout after taking account into the age on four subscales of QoL [15,27,33]. To assess the model fit to the data, an F-test was performed with adjusted R2 values for each regression model. The significance level used for inclusion of covariates in the regression models was set at *p* < 0.05. No interactions terms were entered in the regression models because the age was the only variable that met the criteria to be controlled in the models.

Finally, two one-way multivariate analyses of variance (MANOVA) were used to examine age-based group differences (young versus middle-aged women) among the independent variables and dependent variables. An alpha level of 0.05 was used to determine statistical significance. The effect sizes (ES) were calculated by using the Cohen’s delta (0.2 or less is a small ES, about 0.5 is a moderate ES, and 0.8 or more is a large ES).

## 3. Results

### 3.1. Descriptive Analysis, Scale Reliability, and Correlations

Occupational stress of working women in Shanghai was relatively high in this study, above the mid-point of the stress level (Table 1). Participants were not at risk of burnout, and also had relatively high levels of QoL (physical health, psychological health, social relationship, and living environment) in the present study. Furthermore, internal consistency reliability coefficients and CR for occupational stress, emotional exhaustion, cynicism, reduced professional efficacy, physical health, psychological health, and environment represented acceptable levels of reliability [9], except for social relationship.

The results of the CFA revealed a good fit to the data (χ^2^/df = 32.70/18 = 1.82; IFI = 0.99; CFI = 0.99; RMSEA = 0.047; 90% CI (.019, 0.072)), which indicates acceptable validities of the study variables. Correlation analyses demonstrated significant positive associations between occupational stress and the three subscales of burnout (all *p* < 0.01). Occupational stress was negatively associated with all subscales of QoL, including physical health, psychological health, social relationship, and environment (all *p* < 0.01). Emotional exhaustion, cynicism, and reduced professional efficacy within the burnout construct were significantly negatively related to physical health, psychological health, social relationship, and environment (all *p* < 0.01). Four dimensions of QoL (physical health, psychological health, social relationship, and environment) were positively related to one another in this study. Finally, age was negatively related to occupational stress and the three subscales of burnout (all *p* < 0.01), but was positive associated with all subscales of QoL, including physical health, psychological health, social relationship, and environment (all *p* < 0.01).

### 3.2. Hierarchical Regressions

The assumption of non-multicollinearity was met, since all study variables (VIFs = variance inflation factor) were close to 1 and tolerance statistics were greater than 0.20. Subsequently, four hierarchical multiple regression analyses were performed to determine the role of age, occupational stress, and burnout (i.e., emotional exhaustion, cynicism, and reduced professional efficacy) in explaining the four dimensions of QoL (physical health, psychological health, social relationship, and environments) among working women in Shanghai, and the results are presented in Table 2.

It was found that occupational stress, emotional exhaustion, cynicism, and reduced professional efficacy emerged as significant correlates of participants’ physical health and psychological health (all *p* < 0.05). Social relationship of QoL was predicted by occupational stress (β = − 0.18, *p* < 0.01), emotional exhaustion (β = − 0.14, *p* < 0.05), and cynicism (β = − 0.21, *p* < 0.01), respectively. Both occupational stress (β = − 0.17, *p* < 0.01) and emotional exhaustion (β = −0.18, *p* < 0.01) were significant correlates of participants’ living environment of QoL in this study.

### 3.3. Age-Group Differences

To determine group differences among the study variables, the one-way multivariate analysis of variance (MANOVA) was performed for dependent and independent variables, respectively. A significant main effect (Wilks’ Lambda = 0.73, F (4, 370) = 34.18, *p* < 0.01, ES = 0.27) was observed (Table 3) among the independent variables. Post hoc tests produced significant age effects on occupational stress (F = 53.86, *p* < 0.01, ES = 0.13); emotional exhaustion (F = 80.19, *p* < 0.01, ES = 0.18); cynicism (F = 90.28, *p* < 0.01, ES = 0.20); and reduced professional efficacy (F = 54.79, *p* < 0.01, ES = 0.13). Young women presented higher levels of occupational stress, emotional exhaustion, cynicism, and reduced professional efficacy than middle-aged women.

The second one-way MANOVA was conducted including the four dimensions of QoL as dependent variables. A significant main effect was observed in the model (Wilks’ Lambda = 0.91, F (4370) = 9.03, *p* < 0.01, ES = 0.09) (Table 3). Post hoc tests produced a significant age effect on physical health (F = 24.72, *p* < 0.01, ES = 0.06); psychological health (F = 25.61, *p* < 0.01, ES = 0.06); social relationship (F = 28.32, *p* < 0.01, ES = 0.07); and environment (F = 20.20, *p* < 0.01, ES = 0.05). Overall, young women demonstrated lower physical health, psychological health, social relationship, and environment values compared with middle-aged women in the present study.

## 4. Discussion

Guided by the QoL construct and Lubans et al.’s [26] conceptual framework, the major purpose of this study was to investigate the roles of individual factors (i.e., age) and psychosocial factors (i.e., occupational stress, three subscales of burnout) on four dimensions of QoL among working women in Shanghai. The second purpose was to examine the age differences among study variables. The findings of this study indicated that occupational stress and burnout were significantly associated with QoL among these participants. Two one-factor MANOVAs demonstrated that young working women had higher occupational stress and burnout, but lower levels of QoL than middle-aged women.

Consistent with our first hypothesis, strong negative relationships between occupational stress and the four subscales of QoL were found in this study. The results of the regression models demonstrated that occupational stress consistently emerged as a significant predictor of four dimensions of QoL, regardless of age, in this study. Previous research [36] noted that role proficiency, workload, work environment, and responsibility are important correlates of occupational stress. Thus, the findings of this study suggest that reinforcing occupational skills training, improving the work-related environment, reducing workload, and developing a positive attitude toward the job may be effective ways to improve overall QoL among this population [25,36]. In line with our first hypothesis and a previous study [37], our findings indicated that burnout was associated with occupational stress and psychosocial problems that may facilitate the understanding of burnout-related construct. The main contribution of this study was to expand previous limited evidence related to the relationship of job burnout with QoL among female workers after taking account of the occupational stress level of each female participant. The statistical analyses indicated the three subscales of burnout explained significant variance in QoL after controlling for age. It seems that working women in this study who encountered higher occupational stress from work may have become unmotivated and devalued their work, which may be negatively related to burnout and their QoL. A recent study [20] provided the primary evidence that both occupational stress and job burnout were negative correlates of QoL among nurses in China. The findings of the present study provide additional evidence that the relationship between job burnout and QoL may be domain-specific, given that emotional exhaustion was a consistent predictor of QoL in this study. This is one of the first studies that has investigated the combined effect of both occupational stress and job burnout on QoL among working women in Shanghai regardless of age; therefore, further investigation is needed to confirm these findings.

Consistent with our second hypothesis and previous literature [38], the results demonstrated the significant age differences in occupational stress, emotional exhaustion, cynicism, and reduced professional efficacy in this study. It was found that young working women had higher occupational stress and psychological job demand compared with their counterparts (middle-aged women), and this trend deteriorated with age. These findings indicate that younger women may lack occupational experiences and social support to cope with various aspects of occupational stress, which often lead to job dissatisfaction, job strain, and increased levels of burnout [4,39]. Our results also reflect a potential increase in mental health problems among young working women. Furthermore, young working women often have more aspirations for achievements than middle-aged women. When their achievements are unable to meet their expectations, they often experience high levels of dissatisfaction [40], which suggests that reinforcing occupational skills training, improving the work-related environment, reducing workload, and developing a positive attitude toward the job are effective ways for working women to lower their occupational stress and burnout, and improve their QoL [12,41]. Thus, it will be useful for working women to improve their self-efficacy and abilities to cope with stressors at home and work, and find effective ways to reduce stress levels, such as physical activity [42,43,44,45]. In addition, developing a proactive attitude toward the job, being physically active to prevent and control chronic diseases, and fostering a strong social support system are likely related to decreased occupational stress and burnout, and in turn, increased QoL among working women [5,8,25].

In the current study, the young women reported lower QoL (i.e., physical health, psychological health, social relationship, and environment) compared to their older working women counterparts. Due to the high negative correlation between occupational stress and QoL, this might be one reason young women demonstrated lower QoL, because they had higher occupational stress levels compared to their middle-aged peers. It could also be that family barriers (i.e., married and need to take care of family and kids) are preventing young women from participating in exercise for enhancing their physical health and psychological health, and engaging in social interaction with their peers and friends in/out of the working place, since all of our participants were married working women. In contrast, middle-aged working women may be able to get more support from significant others, have more time and energy, and can develop more working experiences and coping strategies than young women [43]. Based on their sound status and social relationships, better working environment, and experiences, middle-aged working women may feel more confidence to promote their physical health, psychological health, social activities/engagement, and living environment than their young counterparts, and consequently may have more meaningful and positive associations with their health-related behaviors and well-being [43,45]. The findings of this study suggest that interventions aimed to improve working women’s perceived abilities to cope with occupational stress from home and work, such as regular exercise and developing strong social support, are likely related to lower occupational stress and burnout and higher QoL.

The findings of the present study must be considered within the context of inherent limitations. First, the design of this study was cross-sectional, thus causal relationships cannot be established. We recommend continued attentions to interactive relationships among occupational stress, burnout, and QoL in working women using longitudinal and experimental studies to test for causality. Participants in this study were from an urban city in China, producing a sample with high socioeconomic status. We acknowledge the potential selection and participation bias due to the self-selective and non-representative nature of the sample in this study, which may reduce the generalizability of the findings. It is important that future studies should obtain a random sample from multiple cities to increase the generalizability of the findings. In addition, future studies may also consider controlling for other individual factors, such as years of working experience, occupation, and marital status. Although participants were encouraged to answer the questions truthfully and independently, the potential for social desirability response bias and measurement errors was inevitable, especially for self-report measures (i.e., WHOQOL-BREF), which is considered one of the limitations of this study. Furthermore, the use of the long version of WHOQOL-100 could provide detailed information on QoL among working women. To this end, future research is needed to use objective measures such as direct observation and qualitative methods to obtain detailed information in an attempt to understand the underlying correlates of QoL for typical working women, a vital aspect of public health.

## 5. Conclusions

Guided by the QoL construct and Luban et al.’s conceptual framework, understanding how interactions between individual factors (i.e., age) and psychosocial factors (i.e., stress and burnout) may influence mental health outcomes such as QoL is needed and important. The findings of this study suggest ways in which QoL may be enhanced in working women. Working women who are suffering from occupational stress and burnout may perceive a low level of QoL as a vulnerable population, especially among young women. A positive occupational climate and stress coping can contribute to better physical and psychosocial health among this vulnerable population. Occupational therapists can contribute to the reduction of occupational stress and burnout in working women, particularly among young working women, by instigating preventive occupational therapy. Some preventive strategies, such as occupational retreats, building supportive social relationships, and participating in regular leisure-time physical activities with significant others, are recommended.

## Figures and Tables

**Table 1:** Descriptive statistics, internal reliabilities, and correlations among variables (*N* = 375).

Variables		Range	M ± SD	CR	1	2	3	4	5	6	7	8	9
1	Occupational Stress	0–4	2.65 ± 0.62	0.95	(0.95)								
2	Emotional exhaustion	1–7	3.11 ± 1.17	0.75	0.46 **	(0.75)							
3	Cynicism	1–7	2.95 ± 1.27	0.71	0.44 **	0.67 **	(0.71)						
4	Reduced efficacy	1–7	3.53 ± 1.16	0.75	0.18 **	0.39 **	0.47 **	(0.74)					
5	Physical health	4–20	14.41 ± 2.78	0.74	−0.42 **	−0.47 **	−0.51 **	−0.33 **	(0.74)				
6	Psychological health	4–20	14.37 ± 2.74	0.71	−0.41 **	−0.45 **	−0.50 **	−0.32 **	0.72 **	(0.71)			
7	Social relationship	4–20	14.29 ± 2.95	0.52	−0.37 **	−0.41 **	−0.44 **	−0.28 **	0.62 **	0.58 **	(0.75)		
8	Environment	4–20	13.66 ± 2.43	0.71	−0.34 **	−0.39 **	−0.36 **	−0.26 **	0.62 **	0.68 **	0.52 **	(0.71)	
9	Age	18–59	42.06 ±10.58	-	−0.40 **	−0.39 **	−0.39 **	−0.27 **	0.24 **	0.26 **	0.26 **	0.26 **	-

Note: Cronbach’s alpha coefficients are provided along the diagonal in parentheses; M = mean; SD = standard deviation; CR = competitive reliability; ** *p* < 0.01.

**Table 2:** Results of four hierarchical regressions for QoL (*N* = 375).

Dependent Variable	Step #	Independent Variable	R^2^	*F*	β	t
Physical health	Step 1		0.06	23.23 **		
	Step 2		0.33	37.31 **		
		Age			−0.05	−0.96
		Occupational stress			−0.23 **	−4.47
		Emotional exhaustion			−0.17 **	−2.79
		Cynicism			−0.26 **	−4.25
		Reduced efficacy			−0.12 *	−2.48
Psychological health	Step 1		0.07	27.01 **		
	Step 2		0.31	34.24 **		
		Age			−0.01	−0.26
		Occupational stress			−0.22 **	−4.31
		Emotional exhaustion			−0.12 *	−1.96
		Cynicism			−0.28 **	−4.54
		Reduced efficacy			−0.11 *	−2.14
Social relationship	Step 1		0.06	26.08 **		
	Step 2		0.24	22.27 **		
		Age			0.02	0.42
		Occupational stress			−0.18 **	−3.35
		Emotional exhaustion			−0.14 *	−2.22
		Cynicism			−0.21 **	−3.26
		Reduced efficacy			−0.09	−1.74
Environment	Step 1		0.07	27.48 **		
	Step 2		0.19	18.69 **		
		Age			0.06	1.13
		Occupational stress			−0.17 **	−3.07
		Emotional exhaustion			−0.18 **	−2.78
		Cynicism			−0.10	−1.45
		Reduced efficacy			−0.09	−1.73

Note: F-test and adjusted R^2^ were conducted to assess model fit. R^2^ values are cumulative, with each incremental step adding to the variance explained; β values are standardized regression coefficients of the regression analysis. # = number; * *p* < 0.05; ** *p* < 0.01.

**Table 3:** One-way multivariate analysis of variance (MANOVA) (*N* = 375).

Variables	Young Women		Middle-Aged Women		*F* Value
	(*n* = 195)		(*n* = 180)		
	*M*	SR	*M*	SR	
Dependent variables					
1. Occupational Stress	2.86	0.04	2.42	0.04	53.86 **
2. Emotional exhaustion	3.59	0.08	2.6	0.08	80.19 **
3. Cynicism	3.49	0.08	2.36	0.09	90.28 **
4. Reduced efficacy	3.93	0.08	3.1	0.08	54.79 **
Wilks’ Lambda = 0.73; F = 34.18 (4, 370), *p* < 0.01; Partial Eta Squared = 0.27					
Dependent variables					
5. Physical health	13.75	0.19	15.13	0.20	24.72 **
6. Psychological health	13.7	0.19	15.09	0.20	25.61 **
7. Social relationship	13.54	0.2	15.1	0.21	28.32 **
8. Environment	13.13	0.17	14.23	0.18	20.20 **
Wilks’ Lambda = 0.91; F = 9.03 (4, 370), *p* < 0.01; Partial Eta Squared = 0.09					

Notes: M = estimated marginal mean; SR = standard error; ** *p* < 0.01; age range for young women = 18-44; age range for middle-aged women = 45-60.
